# A drop-out mechanism for active learning based on one-attribute heuristics

**DOI:** 10.3389/frai.2025.1562916

**Published:** 2025-09-02

**Authors:** Sriram Ravichandran, Nandan Sudarsanam, Balaraman Ravindran, Konstantinos V. Katsikopoulos

**Affiliations:** ^1^Department of Management Studies, Indian Institute of Technology Madras, Chennai, Tamil Nadu, India; ^2^Department of Data Science and AI, Indian Institute of Technology Madras, Chennai, Tamil Nadu, India; ^3^Wadhwani School of Data Science and AI, Indian Institute of Technology Madras, Chennai, Tamil Nadu, India; ^4^Department of Decision Analytics and Risk, University of Southampton Business School, Southampton, United Kingdom

**Keywords:** active learning, human-in-the loop, human behavior, biases, fast and frugal heuristics

## Abstract

Active Learning (AL) leverages the principle that machine learning models can achieve high accuracy with fewer labeled samples by strategically selecting the most informative data points for training. However, when human annotators provide these labels, their decisions might exhibit a systematic bias. For example, humans frequently rely on a limited subset of the available attributes, or even on a single attribute, when making decisions, as when employing fast and frugal heuristics. This paper introduces a mathematically grounded approach to quantify the probability of mislabeling based on one attribute. We present a novel dropout mechanism designed to influence the attribute selection process used in annotation, effectively reducing the impact of bias. The proposed mechanism is evaluated using multiple AL algorithms and heuristic strategies across diverse prediction tasks. Experimental results demonstrate that the dropout mechanism significantly enhances active learning (AL) performance, achieving a minimum 70% improvement in effectiveness. These findings highlight the mechanism's potential to improve the reliability and accuracy of AL systems, providing valuable insights for designing and implementing robust intelligent systems.

## 1 Introduction

Prediction models are integral to automating decision-making processes across diverse industries, enabling organizations to make data-driven, efficient, and informed choices instead of relying only on intuition or past experiences. In numerous practical settings, obtaining labels for data instances is significantly more resource-intensive than acquiring their input attribute values. For example, in healthcare, a hospital must identify which patients require intensive care. In banking, financial institutions need to determine which customers qualify for loan approvals. Similarly, in recruitment, IT firms aim to shortlist candidates suitable for specific roles.

In each case, collecting attribute information (e.g., medical records, financial history, or resume details) is relatively straightforward, while obtaining accurate labels (e.g., critical care necessity, loan eligibility, or applicant suitability) involves higher costs, effort, or expertise.

Active Learning (AL) offers a powerful approach to address such challenges by strategically selecting data points to be labeled. This enables benchmark predictive accuracy to be achieved with significantly fewer labeled instances.

**Figure 1:**
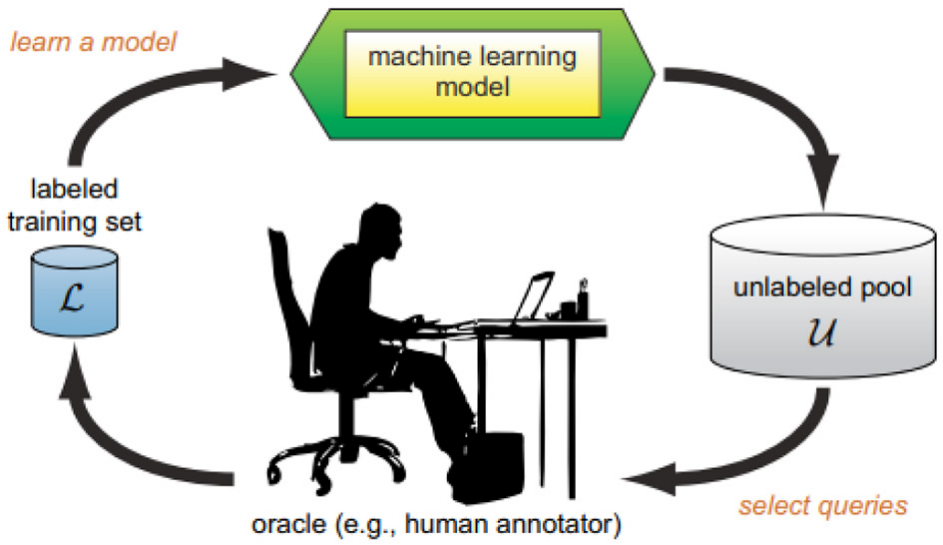
A typical active learning cycle as described by (Settles 2009).

The Active Learning (AL) cycle, depicted in Figure 1, generally begins with a small set of labeled instances, referred to as L. This initial set is used to train a standard supervised machine learning model. Subsequently, one or more instances are strategically sampled from the unlabeled pool U without replacement for querying. The labels obtained from the oracle, along with the information regarding the queries, are then used to augment the labeled set. This updated set is employed to retrain the machine learning model, continuing the cycle.

This approach enables predictive models to achieve better performance more efficiently, using fewer labeled data points, making it particularly valuable in scenarios where labeling is costly or challenging (Settles, 2009; Monarch, 2021; Amin et al., 2023; Robinson et al., 2024). Many previous studies in Active Learning assume the oracle to be an unbiased annotator (Venugopalan and Gupta, 2022; Arora et al., 2009; Guo and Schuurmans, 2007).

However, many of these applications rely on Human-in-the-Loop (HITL) systems, where human annotators play a pivotal role in labeling and decision-making processes. For instance, medical diagnoses are made by doctors, while decisions on loan applications are handled by bank managers, among other examples. Consequently, the labels provided to an Active Learner may reflect such biases, potentially reducing the performance of the trained model (Agarwal et al., 2022a). More specifically, annotators may rely on fast-and-frugal heuristics when providing labels to the Active Learner (Todd et al., 1999). Psychological research has shown that humans often exhibit bias when making decisions (Ariely, 2010), even if ultimately the decisions might be as accurate as those made by more complex and supposedly more rational models (Gigerenzer et al., 2011; Katsikopoulos et al., 2020). The work by (Anahideh et al. 2022) introduced a fair active learning framework that incorporates fairness into the sampling process, selecting data points for labeling in a way that balances model accuracy with algorithmic fairness. However, this approach does not consider the heuristics involved in human decision-making and only focuses on ensuring algorithmic fairness in predictions rather than addressing fairness in the labels provided by the annotators.

In a management context, automating the decision-making process of hiring managers often involves using an active learning approach. In this setup, the active learner selects candidate profiles (i.e., instances to be labeled) and queries an experienced hiring manager for their hiring decisions (labels). However, hiring managers frequently rely on heuristics, such as favoring candidates from prestigious universities or prioritizing resumes with specific key terms. While these features are easily recognizable, they may not accurately predict job performance. This reliance on heuristics can introduce bias into the provided labels, and when such biased labels are used to train machine learning models, they can compromise the overall quality and fairness of the model's decisions.

(Ravichandran et al. 2024) explored the impact of human heuristics on Active Learning algorithm performance and proposed a novel query strategy that prioritizes points likely to yield accurate labels.

Our work extends beyond reordering queries by introducing a drop-out mechanism designed to mitigate mislabeling. The approach involves first estimating the probability of a data point receiving an incorrect label based on using one attribute. The mechanism then identifies attributes with a high likelihood of introducing bias and excludes their information during querying. By preventing annotators from basing their decisions on these attributes, the mechanism reduces the chances of incorrect labeling and enhances the reliability of the provided labels.

The proposed drop-out mechanism has broader implications for human-in-the-loop systems. Effectively managing the information presented to annotators not only enhances label accuracy but also reduces the cognitive load on human oracles. This approach can be applied in diverse domains where labeling is inherently subjective or prone to bias, such as legal document classification, financial risk assessment, and social media content moderation, thereby improving the reliability of machine learning models in these areas.

A preliminary version of this work was accepted as a short paper in the Second Workshop on Hybrid Human-Machine Learning and Decision Making at the European Conference on Machine Learning and Principles and Practice of Knowledge Discovery in Databases (ECML PKDD'24), However this study has been significantly expanded. The current version incorporates the Fast and Frugal Tree heuristic alongside Take the Best, integrates the information density query strategy with entropy sampling, and extends experiments to ten datasets. Moreover, we also include a detailed analysis of the mechanism's relative effectiveness across various heuristics and active learning algorithms. All sections have been thoroughly revised and enhanced to provide deeper insights into the study.

The paper is organized as follows: Section 2 reviews related literature pertinent to our study. Section 3 outlines the methodology, detailing the AL algorithms, the proposed drop-out mechanism, and the fast-and-frugal heuristics considered. This is followed by the Results and Discussion section, which includes both the mathematical framework and experimental findings supporting the proposed mechanism. The subsequent section discusses the limitations of the study and outlines directions for future research. The paper concludes with a summary of key findings and contributions in the Conclusion section.

## 2 Related work

Active learning (AL) algorithms in this study are restricted to a pool-based sampling scenario, wherein a small set of labeled data points exists, and the remaining unlabeled data is available all at once. These algorithms can be broadly categorized into two groups. The first group ranks data points uncertainty or entropy metrics (Shannon, 1948). Labels are queried sequentially based on the rankings generated across the pool of unlabeled data points. Despite their apparent simplicity, such methods often demonstrate competitive performance (Raj and Bach, 2022; Liu and Li, 2023). The second group extends beyond ranking by incorporating spatial information of data points, combining uncertainty with *information density*. Information density measures how representative an unlabeled data point is of the overall distribution, and the two measures are often combined multiplicatively to enhance query effectiveness (Settles and Craven, 2008).

Many AL studies implicitly assume an oracle that is bias-free (Hoi et al., 2006; Arora et al., 2009; Guo and Schuurmans, 2007). However, empirical evidence challenges this assumption. For instance, (Baum and Lang 1992) demonstrated that incorporating human oracles in query learning can lead to a significant increase in generalization errors, up to fortyfold. This underscores the vulnerability of AL performance in the presence of biased or erroneous human annotators. Furthermore, psychological research has established that human decision-making often relies on heuristics, which could lead to systematic bias in the labels provided to active learners (Agarwal et al., 2022a).

Research on human biases in AL has shed light on their adverse impact and proposed methods for mitigation. Behavioral biases were found to reduce classification accuracy by at least 20% in specific datasets (Agarwal et al., 2022b). The study *Active Learning with Human-Like Noisy Oracle* modeled human oracles where noise levels varied with oracle confidence. Their proposed algorithm effectively addressed example-dependent noise, outperforming traditional uncertainty-based methods (Du and Ling, 2010). Similarly, (Groot et al. 2011) employed a Gaussian Process framework to capture annotator expertise and manage disagreements, significantly improving regression tasks involving noisy, subjective labels.

Several studies have refined AL techniques by incorporating user feedback or personalizing learning processes. For example, repeated-labeling strategies demonstrated improvements in label quality and model performance in noisy environments, while personalized active learning for collaborative filtering tailored queries to user preferences, thereby improving recommendation accuracy (Harpale and Yang, 2008). Extensions to traditional AL frameworks, such as incorporating feature importance feedback, have shown promise in accelerating learning for applications like news filtering (Raghavan et al., 2006). Additionally, evidential uncertainty sampling strategies grounded in belief function theory have effectively balanced exploration and exploitation, outperforming traditional uncertainty sampling approaches (Hoarau et al., 2024).

In the realm of human decision-making, the concept of fast-and-frugal heuristics is a central theme in behavioral science literature. These heuristics describe how individuals rely on a limited subset of attributes to make decisions quickly and efficiently without considering all available information. Unlike other approaches to studying heuristics, this approach has been instrumental in developing mathematical models (Katsikopoulos et al., 2020) that provide a structured framework for understanding how people estimate quantities, choose between multiple options, or classify objects into categories based on simplified decision rules.

The validity of these heuristics has been supported by extensive empirical studies (Gigerenzer et al., 2011), which demonstrate their applicability and robustness in real-world scenarios. For instance, research by (Katsikopoulos et al. 2020) highlights how these heuristics can achieve remarkable accuracy across diverse decision-making contexts, often rivaling more complex algorithms. Furthermore, the work by (Todd et al. 1999) underscores the close alignment between heuristic-based decision-making and actual human behavior, reinforcing the idea that these simplified strategies are not only efficient but also deeply rooted in natural human cognition.

These findings suggest that fast and frugal heuristics are not just a compromise for limited cognitive resources but are adaptive strategies optimized for various environments, enabling individuals to make effective decisions with minimal effort. These heuristics suggest that human annotators might focus on a particular set of attributes, potentially neglecting others, when labeling data. Despite its relevance, existing AL research largely overlooks the implications of such heuristics for system design.

The study by (Ravichandran et al. 2024) represents one of the few efforts to address human heuristics in AL. They proposed a novel query strategy that prioritizes data points less susceptible to labeling bias. While this method reduces the impact of biased labels on AL effectiveness, it does not mitigate the bias inherent in the labels obtained for queried instances. (Raghavan et al. 2006) addressed the cognitive challenges faced by annotators by simplifying queries through feature subsetting. Their approach, which combines class label queries with feature feedback, significantly improved classifier performance over soliciting class labels alone.

In summary, while the influence of incorrect labeling by human annotators on AL performance is increasingly recognized, notable gaps remain. Limited research on human heuristics in active learning primarily seeks to mitigate the effects of biased labels in AL through modified data selection strategies or feedback mechanisms. However, directly addressing and preventing the occurrence of biased labels from human annotators continues to be a significant challenge in AL research.

## 3 Methodology

**Figure 2:**
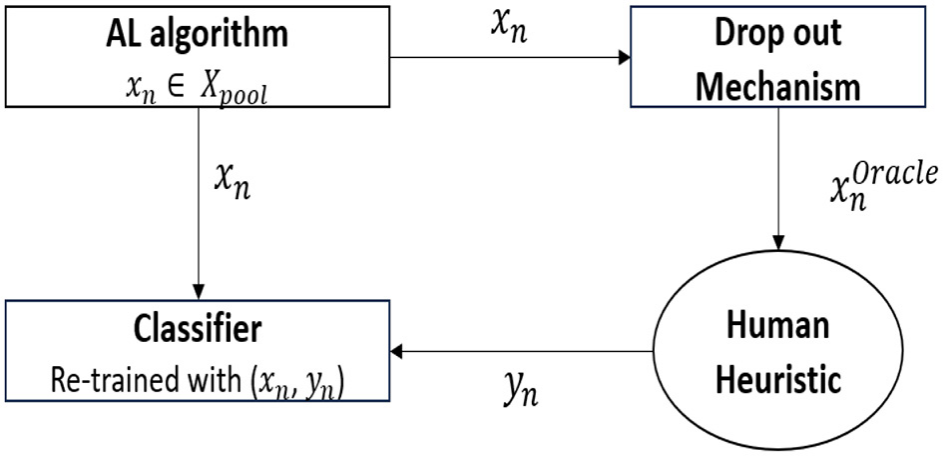
Methodological framework.

As shown in Figure 2, the methodological framework includes a standard active learning algorithm(entropy sampling) that chooses the data point(*x*_*n*_) from a pool of unlabeled data points to query(*X*_*pool*_). From the data point chosen, the proposed drop-out mechanism drops the attribute values that must not be presented to the oracle. This results in $x_{n}^{oracle}$, which contains a subset of attributes present in *x*_*n*_ that are to be sent to the oracle for labeling. In this study, we mimic the functionality of an oracle by using fast and frugal heuristics, such as Take the best(TTB) and Fast and Frugal Tree (FFT), as the decision strategies used by the oracle. The heuristic finally provides the label(*y*_*n*_), which is then used to train the underlying classifier.

We further explain the methodological framework using a use-case scenario where a doctor is tasked with diagnosing the presence of a disease on a pool of patient records. We now aim to build a prediction model that automates this decision. Typically, an active learning (AL) algorithm chooses the most uncertain or informative record from the pool to query the doctor.

Once the record (*x*_*n*_) is selected, the novel drop-out mechanism proposed in this study removes certain attributes before presenting the record to the doctor (the oracle). These dropped attributes may be important, but they are excluded to prevent the doctor from making an incorrect diagnosis.

The doctor then provides a label (*y*_*n*_), which is used to update the classifier. After receiving the label, the classifier is retrained using all the attribute information, including both the newly labeled data and the existing records. This cycle is repeated.

The following subsections provide an overview of the proposed dropout mechanism and active learning algorithms, along with a description of the heuristics used to model the synthetic oracle.

### 3.1 Overview of the proposed mechanism

The quality of a label assigned to a query depends on the attribute selected by the heuristic for labeling. This selection process can be influenced by deliberately concealing certain attribute information during querying. Drawing inspiration from the dropout technique in deep learning, which involves randomly dropping nodes to prevent overfitting (Srivastava et al., 2014), we propose a similar approach where attributes are selectively excluded during querying.

To determine which attributes to drop for each query, we use the following formulation, which calculates the probability that a queried data point would receive an incorrect label based on the absolute deviation of the data-points attribute values from the population median (a, b):


(1) $$\begin{array}{l} P_{\text{mislabelling}}\left(a,b\right)=0.5-\frac{1}{90}\tan^{-1}\frac{\min\left(a,b\right)}{\max\left(a,b\right)} \end{array}$$

The derivation of this formulation is elaborated upon in the subsequent section. The proposed algorithm is designed to refine a queried instance *X*_*n*_ by systematically dropping attributes based on their likelihood of contributing to mislabelling. The process begins by initializing an empty list *DropList* to store the attributes to be removed and another list to record the mislabelling probabilities for all candidate attribute pairs. These probabilities reflect the likelihood of mislabelling when an attribute is utilized in decision-making. All possible attribute pairs from *X*_*n*_ are considered for evaluation.

For each pair, the absolute differences between the values of the attributes in *X*_*n*_ and their respective medians in the entire dataset *X* are calculated. Using these differences, the likelihood of mislabelling for each pair is computed based on Equation 1. For each pair, the attribute with a smaller deviation from its median is identified as a candidate for removal if the mislabelling probability exceeds a threshold of 0.3.

Once all pairs have been evaluated, the mislabelling probabilities are sorted in descending order, and the corresponding attributes are updated in *DropList*. After removing duplicates, the first two attributes in *DropList* are selected for removal from the queried instance, resulting in the refined query *X*_*oracle*_. This refinement ensures that the heuristic is prevented from relying on the removed attributes during the decision-making process, thereby improving the overall robustness of the system. The pseudo-code of the mechanism is followed (Algorithm 1).

**Algorithm 1:** Proposed drop-out mechanism

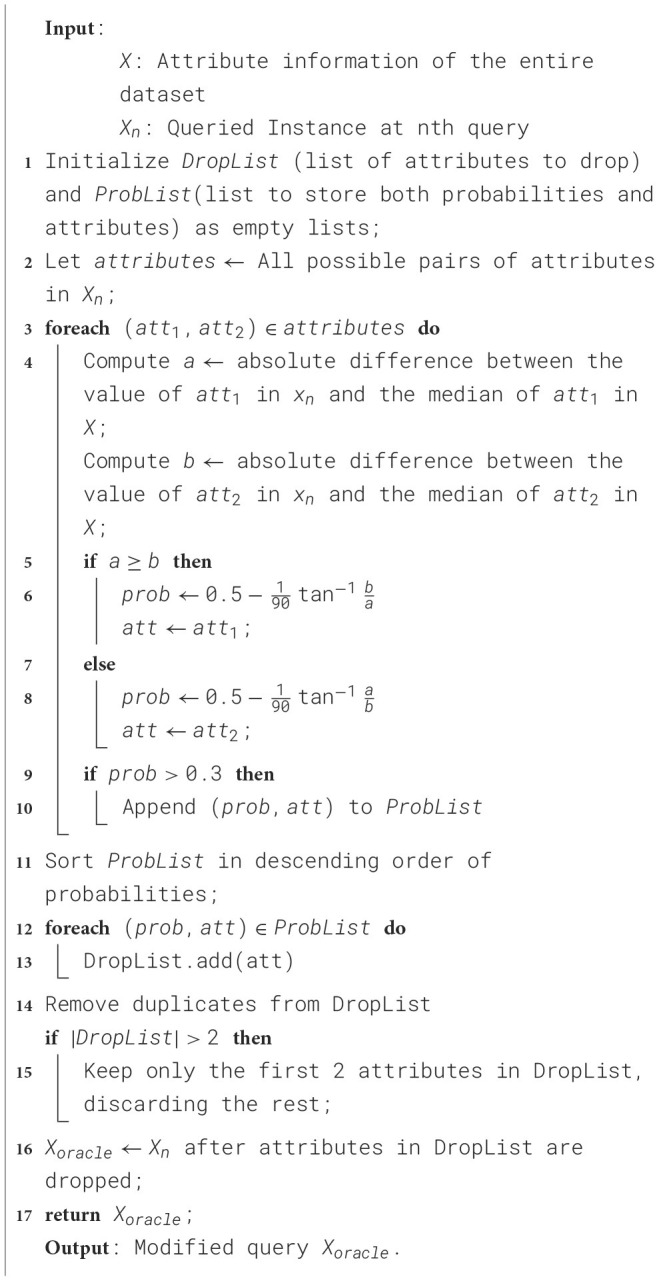

### 3.2 Active learning algorithms

In this study, we examine two active learning (AL) algorithms: Entropy Sampling and Information Density. Each algorithm represents one of the two categories outlined in Section 2, covering the key variations in widely used standard AL algorithms.

Entropy Sampling selects the data point with the highest uncertainty(E(x)), as measured by the following expression:


(2) $$\begin{array}{l} x^{*}=\arg\underset{x\in X_{U}}{\max}-\overset{K}{\underset{i=1}{\sum }}p_{C}\left(y_{i}/x\right)\log\left(p_{C}\left(y_{i}/x\right)\right) \end{array}$$

In this equation, *p*_*C*_(*y*_*i*_/*x*) denotes the probability that data point *x* belongs to class *y*_*i*_, considering *K* possible label assignments.

On the other hand, the Information Density algorithm takes into account the spatial position of each data point in the feature space. It computes the average Euclidean similarity between the data point in question and all other points, which leads to the following optimization problem:


(3) $$\begin{array}{l} x^{*}=\arg\underset{x\in X_{U}}{\max}\left[E\left(x\right)\cdot \frac{1}{U}\underset{x_{u}\in X_{U}}{\sum }\text{sim}\left(x,x_{u}\right)\right] \end{array}$$

### 3.3 Synthetic oracles mimicking human heuristics for labeling queries with dropped attributes

Behavioral science research has identified various heuristics that humans employ when making decisions. For our study, we focus on two widely used and effective heuristics: Take-the-Best (TTB) and Fast-and- Frugal Tree (FFT). These methods are valued for their simplicity, efficiency, and adaptability, making them well-suited for decision-making scenarios. We further explore how these heuristics can serve as the foundation for synthetic oracles designed to label data points, even in the presence of missing attributes.

The TTB heuristic operates by prioritizing the most influential attribute, which humans often perceive as the most decisive. When a data point is queried, TTB evaluates a single feature and assigns a label if its value exceeds the population median. In our implementation, we measure the decision accuracy of each attribute to identify the best-performing one. If the queried instance lacks the most predictive attribute, TTB uses the next best alternative, enabling the oracle to provide labels even when some features are missing. The possibility of information loss due to dropping attributes is factored in by the decrease in quality of labels provided by the human heuristic-based annotator, which picks sub-optimal attributes while providing labels.

For example, consider a loan approval task where the most predictive attribute is the applicant's credit score. If the credit score exceeds the median value, TTB will approve the loan. If the credit score is unavailable, TTB would use the next most informative feature, such as the applicant's annual income, to make the decision.

**Figure 3:**
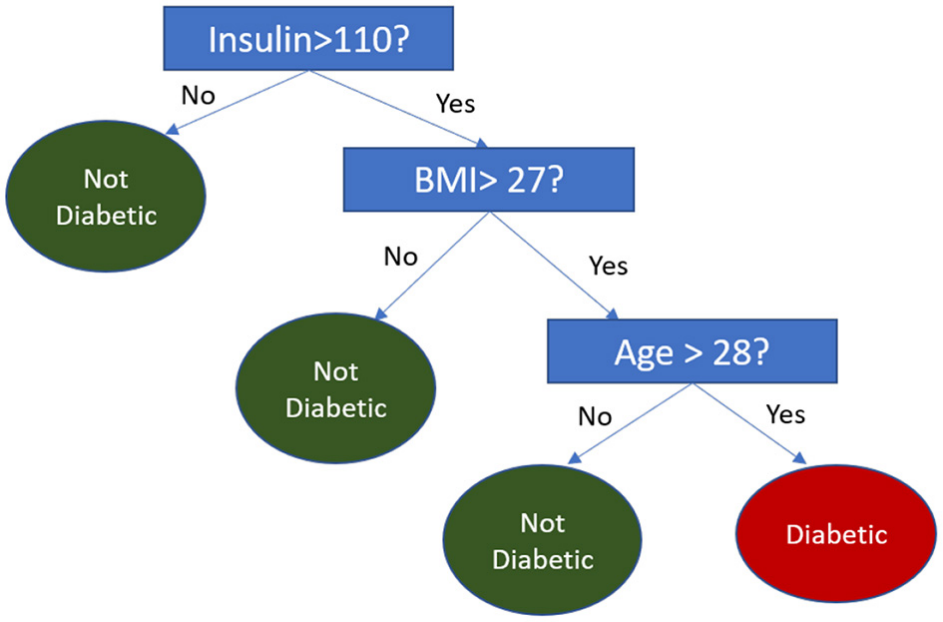
Fast and frugal tree for diabetes prediction.

The FFT heuristic operates using a decision tree structure, where attributes are binarized through median splits. When an attribute required by the FFT is missing in a query, the tree skips that feature and instead uses the remaining attributes to make a decision. Figure 3 demonstrates an example of this process, where, if a person's BMI is unavailable, the decision is based solely on the Insulin and Age attributes.

The above methodologies ensure that the synthetic oracle remains functional and robust, accurately mimicking human decision-making even when attributes are dropped during the querying process.

## 4 Results and discussion

This section begins with a derivation of the mathematical formulation underlying the proposed mechanism. It then presents an extensive empirical evaluation of the mechanism's effectiveness across active learning algorithms and human heuristics, applied to various prediction tasks spanning multiple domains.

### 4.1 Derivation of the mathematical framework behind the dropout mechanism design

**Figure 4:**
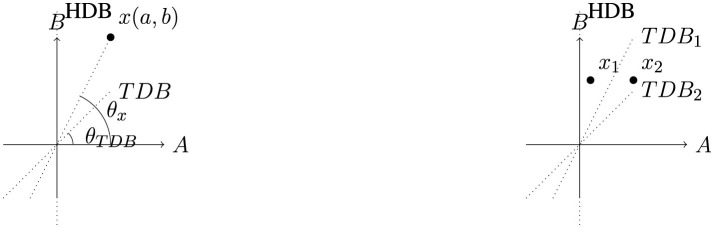
Left: figure describing the notations used in the study. Right: figure showing the intuitive reason behind *x*_1_ having more chances of being mislabeled by heuristic.

Fast-and-frugal heuristics enable human oracles to make decisions based on one or more attributes. Consider a prediction task involving attributes A and B, as depicted in Figure 4. The origin represents the median values, and the Heuristic Decision Boundary (HDB) will align with the A-axis if the heuristic uses attribute A and with the B-axis if attribute B is used. Since the true decision boundary is inherently unknown, we rely on the below assumptions to characterize it:

Assumption 1: The two classes are assumed to be linearly separable, meaning that they can be perfectly separated by a straight line in a 2-dimensional space (or a hyperplane in higher dimensions). Given two attributes, *A* and *B*, the true decision boundary (TDB) is mathematically represented by the linear equation *w*_1_*A*+*w*_2_*B*+*w*_3_ = 0, where *w*_1_, *w*_2_, *w*_3_≥0. A point (*a, b*) lies on one side of this line if it belongs to one class and on the opposite side if it belongs to the other class.

Assumption 2: The true decision boundary (TDB) is assumed to pass through the median values of the attributes, i.e., the origin in Figure 4. It forms an angle θ_*TDB*_ with one of the attribute axes, where θ_*TDB*_ is equally likely to take any value between $\frac{\pi }{4}$ and $\frac{\pi }{2}$.

In Figure 4 (left), let x be a datapoint, making an intercept of a and b with Axes A and B, respectively. Here, θ_*x*_ is the angle made by datapoint x with axis A. θ_*TDB*_ is the angle made by True decision boundary with axis A that ranges between $\frac{\pi }{4}$ and $\frac{\pi }{2}$. The probability of an oracle mislabelling a data point based on the angle it forms with the A-axis (*P*_mislabelling_(θ_*x*_)) can be expressed as follows:


(4) $$P_{\text{mislabelling}}(\theta_{x})=\{\begin{array}{ll} 1, & \text{if }\theta_{TDB}<\theta_{x},\\ 0, & \text{otherwise.} \end{array}$$

As shown in Figure 4 (Right), data point *x*_1_ will be misclassified if the TDB is one of the boundaries *TDB*_1_ or *TDB*_2_. However, *x*_2_ would only be misclassified if the TDB aligns with *TDB*_2_, meaning *x*_1_ is more prone to misclassification compared to *x*_2_. This behavior is mirrored for data points with the key attribute value less than the splitting value. Thus the probability of misclassification for a data point depends on the number of TDB scenarios where the point lies between the TDB and HDB. Hence Equation 3 can be modified as follows:


(5) $$\begin{array}{l} P_{\text{mislabelling}}\left(\theta_{x}\right)=\frac{\int_{\theta_{x}}^{\frac{\pi }{4}}d\theta }{\frac{\pi }{2}} \end{array}$$

We must note that $\theta_{x}=\tan^{-1}\frac{b}{a}$, we rephrase the above equation using attribute values:

The above can thus be shown as:


(6) $$\begin{array}{l} P_{\text{mislabelling}}\left(a,b\right)=0.5-\frac{1}{90}\tan^{-1}\frac{a}{b} \end{array}$$

To bound the equation within 0 and 1:


(7) $$\begin{array}{l} P_{\text{mislabelling}}\left(a,b\right)=\max\left(0,\min\left(1,0.5-\frac{1}{90}\tan^{-1}\frac{a}{b}\right)\right) \end{array}$$

It must be noted that Equation 6 is valid only when the HDB is along B (Attribute A is used by the heuristic). When HDB is along A (Attribute B is used by the heuristic), the $\frac{a}{b}$ will be replaced by $\frac{b}{a}$. This enunciates that when the heuristic picks attribute A in decision-making, the error probability is 0 for data points with *b*>*a* and vice versa.

Since the attribute picked by the heuristic is not known to the active learner, we take the worst-case scenarios to compute the probabilities:


(8) $$\begin{array}{l} P_{\text{mislabelling}}\left(a,b\right)=0.5-\frac{1}{90}\tan^{-1}\frac{min\left(a,b\right)}{max\left(a,b\right)} \end{array}$$

**Figure 5:**
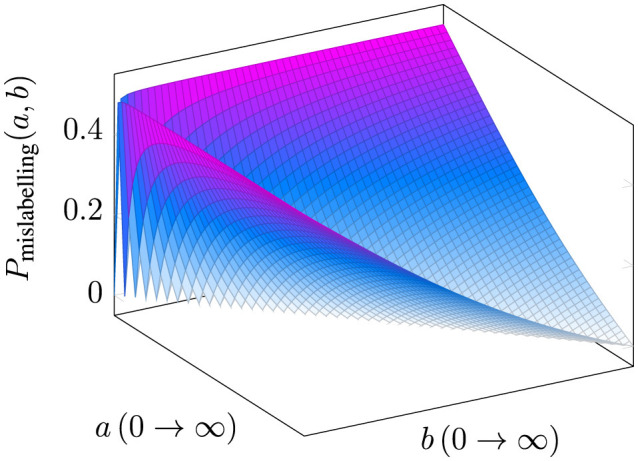
A three-dimensional plot depicting the probability of a data point being misclassified as a function of the deviation values *a* and *b* of the attributes from their median, where *a* and *b* range from 0 to ∞.

**Figure 6:**
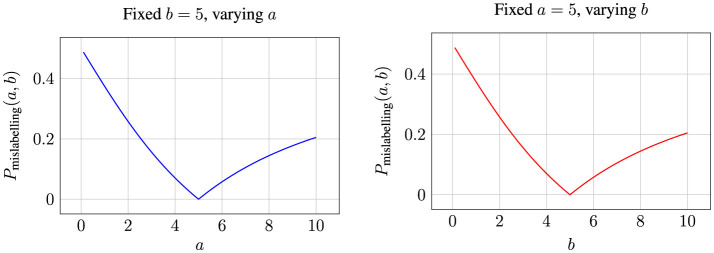
Labeling error probabilities for fixed values of *a* and *b* while varying the other attribute.

Figure 5 illustrates the variation in label error probability based on the values of attributes a and b. To provide a clearer understanding of how the probability values change, we fix the values of each attribute and reduce the plot to two dimensions, as shown in Figure 6.

When the deviation of attribute values from the median is null, i.e., when either *a* or *b* is 0, the probability of mislabeling is at its maximum. This observation aligns with the hypothesis empirically validated in (Ravichandran et al. 2024), where they demonstrate that data points are more likely to be misclassified when the key attribute values are closer to the population median. Furthermore, as illustrated in the figures, the probability of mislabeling decreases as the attribute values deviate further from the median, i.e., as *a* or *b* increases. This probability reaches zero when the deviations of both attributes under consideration are equal. Notably, points with this configuration lie along a 45° line with respect to the attribute axes, implying that no plausible heuristic decision boundary- true decision boundary combination can exist to misclassify such points.

When *a*<*b*, the error probability is influenced by the likelihood of decisions being made based on Attribute *A*, and conversely, when *a*>*b*, the error is driven by Attribute *B*. This behavior is governed by the interaction between the minimum and maximum terms in the equation. As *a* or *b* increases beyond a certain threshold (e.g., 5), the probability of mislabeling gradually rises from zero, reflecting the shifting influence of the relative magnitudes of the attributes on the classification decision.

We further explain the above using a practical scenario, where *a* and *b* represent deviations from standard benchmarks: *a* denotes the deviation of a project's budget from the median budget for similar projects, and *b* represents the deviation of the timeline from the median timeline. When both *a* and *b* are close to zero, the project's attributes align closely with the median values, creating ambiguity in labeling the project as “on track” or “at risk”. This leads to the highest probability of misclassification, as the lack of significant deviations obscures clear decision-making. Conversely, as either *a* or *b* increases, the probability of misclassification decreases. For example, a moderate budget deviation (*a* = 3) with a fixed timeline deviation (*b* = 5) provides additional clarity, indicating whether the project is manageable or at risk. Similarly, larger deviations offer more decisive information for classification.

When *a* and *b* are equal (e.g., both deviations are 10), the project reflects a balanced trajectory of deviations, leading to minimal ambiguity for the heuristic used by the annotator. This symmetry ensures a clear understanding of the project's risk level.

Conversely, when there are disproportionate deviations (*a*>*b*), the focus of classification shifts to the dominant attribute. For instance, a substantial deviation in the budget (*a* = 10, *b* = 2) reduces the likelihood of misclassification based on the budget attribute itself. In this case, any misclassification is more likely to occur due to decisions based on the timeline attribute when assigning labels.

As mentioned in Algorithm 1, the proposed drop-out mechanism removes attributes when the probability of error exceeds 30%, which corresponds to data points that are inclined within 18° of the A or B axis. For example, if data point *x*_1_ is inclined within 18° of the B-axis, attribute A would be dropped during querying, thereby forcing the heuristic to rely on other attributes for decision-making.

Hence, this section provides the derivation of the key formulation behind the design of the proposed dropout mechanism, supporting its relevance.

### 4.2 Experimental evaluation of the mechanism's effectiveness

To assess the effectiveness of the proposed drop-out mechanism, we conducted extensive experiments involving two active learning (AL) algorithms, two fast-and-frugal heuristics, and ten diverse datasets.

All datasets were sourced from the UCI ML Repository (Kelly et al., 2023), spanning multiple domains. A detailed summary of the datasets used has been provided in Table 1. It is worth mentioning that all datasets used in this study have been referenced and analyzed in multiple published works (Jalali et al., 2017; Xie and Braga-Neto, 2019).

**Table 1:** Description of datasets used for the study.

**Sr. No**.	**Dataset name**	**No. of attributes**	**No. of data-points (class ratio)**	**Prediction task**
1	Raisin	7	900 (0.5)	Predict type of raisin (Kecimen or Besni) based on their morphological features
2	Wholesale customer	7	440 (0.67)	Predict customer purchase channel based on annual spending on various products
3	Breast cancer	9	569 (0.63)	Predict the recurrence of tumor based on relevant health information
4	Maternal health	6	200 (0.6)	Predict the risk level of maternal mortality based on Age, BP measures, etc.
5	Car Condition	6	400 (0.5)	Predict the condition of a Car based on its structural features, maintenance, and buying price
6	Diabetes	8	765 (0.65)	To predict whether a patient has diabetes based on patient diagnostic measurements
7	Wine	12	178 (0.67)	Predict the class of Wine based on the quantity of their constituents
8	Chronic Kidney	24	200 (0.63)	Predict the presence of Chronic kidney disease based on Age, RBC count, etc.
9	Audit risk	26	777 (0.61)	To predict whether a firm is fraudulent based on the present and historical risk factors.
10	Algerian Forest	9	244 (0.55)	Predict occurrence of forest fire weather data observations

The performance of AL algorithms is commonly evaluated using learning curves, which plot the number of queried data points against the classification accuracy of a base classifier on a test set. The entire dataset with ground truth labels were used in our study as the test set. The Train set is at first initialized with a small subset of datapoints that are labeled by the heuristic. Later, after every query, the heuristic-labeled datapoint is added to the training set, which is then used to re-train the base classifier after every query. This process is continued until every datapoint is queried.

In this study, we chose logistic regression as the base classifier due to its simplicity and widespread adoption. While more advanced classifiers could potentially achieve benchmark accuracy more quickly, they may significantly shorten the learning phase. This would make it challenging to analyze the differences in learning curves with and without dropout. Moreover, since the primary objective of this study is to facilitate the acquisition of more accurate labels from the oracle–ultimately enhancing model performance—the proposed mechanism remains agnostic to the choice of the base classifier or performance metric considered. Therefore, we limit our experiments to logistic regression.

**Figure 7:**
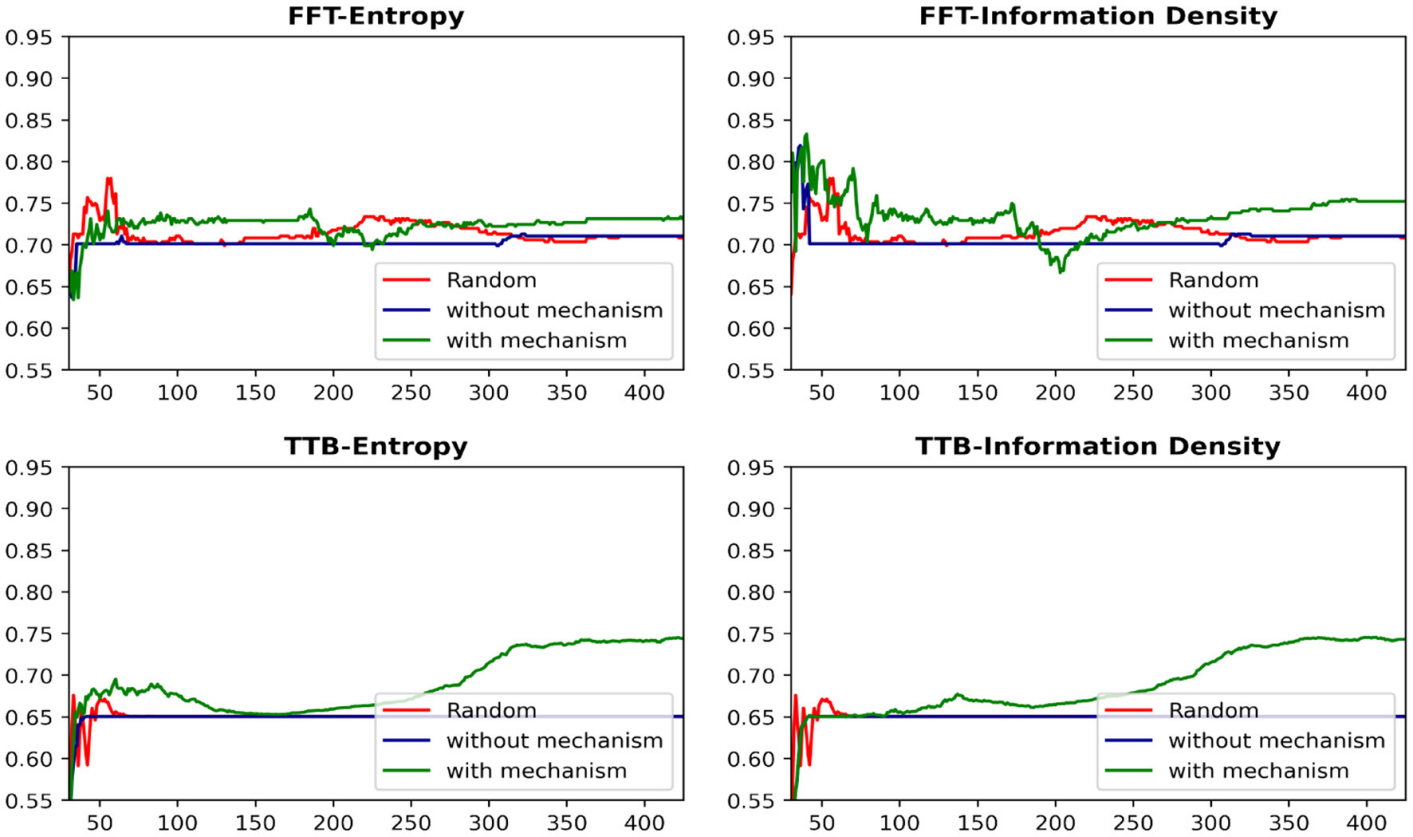
Learning curves showing the effectiveness of dropout mechanism on car dataset. The x-axis represents the number of data-points queried and y axis represents the classification accuracy on the hold-out set.

Figure 7 presents the learning curves for each heuristic-AL algorithm combination on the Car dataset under both conditions. While existing AL algorithms sometimes underperform compared to random sampling, integrating the proposed drop-out mechanism significantly enhances their performance. Most notably, this improvement is reflected in a substantial increase in the area under the learning curve throughout the majority of the querying process.

Table 2 presents the average area under the learning curve across ten iterations for ten different datasets, highlighting the variation in the impact of the drop-out mechanism across various active learning algorithms, datasets, and heuristics.

**Table 2:** Area under the learning curves showing the effectiveness of the drop-out mechanism.

**Dataset**	**Heuristic**	**Random sampling**	**Entropy**		**Information density**	
			**w/o mech**.	**w/ mech**.	**w/o mech**.	**w/ mech**.
Raisin	FFT	619.36	616.97	**620.12**	617.47	**619.27**
	TTB	621.66	623.63	**625.47**	**623.67**	620.47
Wholesale customer	FFT	361.73	346.10	**368.48**	361.89	**366.35**
	TTB	346.15	**370.94**	369.13	361.57	**361.89**
Breast cancer	FFT	468.25	**474.80**	472.04	474.97	474.97
	TTB	506.54	508.13	**508.74**	507.97	**508.63**
Maternal health	FFT	183.81	**178.76**	177.74	**179.99**	179.51
	TTB	189.99	185.28	**191.81**	**190.17**	188.57
Car condition	FFT	301.27	297.09	**305.50**	298.29	**310.85**
	TTB	274.25	274.03	**293.01**	273.82	**292.13**
Diabetes	FFT	510.37	515.16	**534.11**	518.01	**536.33**
	TTB	525.15	524.20	**530.44**	524.52	**531.33**
Wine	FFT	144.15	143.94	**151.08**	143.76	**150.49**
	TTB	164.74	166.15	**166.62**	165.69	**165.78**
Chronic kidney	FFT	138.60	140.89	**140.90**	140.43	**140.95**
	TTB	137.39	140.78	**140.79**	140.41	**140.50**
Audit risk	FFT	677.44	688.49	688.49	688.81	688.49
	TTB	657.44	660.59	660.59	660.42	660.42
Algerian forest	FFT	180.75	**190.87**	190.35	189.58	**189.63**
	TTB	167.78	175.38	**175.49**	175.26	**175.31**

The best-performing values are highlighted in bold.

The proposed mechanism led to a significant improvement in approximately 15 out of 20 dataset-heuristic combinations, regardless of the active learning algorithm used. From a heuristic perspective, the mechanism was effective in 15 scenarios when the TTB heuristic was applied, compared to 13 scenarios with the FFT heuristic. It is important to note that, even in cases where the mechanism did not show a significant improvement–such as in the audit risk scenario–the decrease in performance was minimal.

To provide a comprehensive overview of our analysis, we present Table 3, which averages the performance across all datasets.

**Table 3:** Average area under the learning curves across datasets depicting the effectiveness of the drop-out mechanism.

**Heuristic**	**Random sampling**	**Entropy**		**Information density**	
		**w/o mech**.	**w/ mech**.	**w/o mech**.	**w/ mech**.
FFT	385.57	359.30	**364.88**	361.32	**365.68**
TTB	359.16	362.91	**366.21**	362.35	**364.50**

Bold represents the best performing scenario.

We conducted paired t-tests to evaluate the impact of the dropout mechanism on the area under the learning curves (AULC) for both sampling strategies across 10 datasets. For Entropy Sampling, there was a statistically significant improvement in performance when the dropout mechanism was applied (mean AULC = 365.54) compared to when it was not (mean AULC = 361.11), t(19) = 2.66, *p* = 0.0077. A similar trend was observed for Information Density Sampling, where the application of the dropout mechanism also resulted in a statistically significant performance gain (mean AULC = 365.09 vs. 361.83 without dropout), t(19) = 2.32, *p* = 0.015. These results indicate that incorporating the dropout mechanism consistently enhances the model's learning performance across datasets and sampling strategies.

It is evident that the proposed mechanism consistently led to a significant improvement in performance, irrespective of the active learning algorithm or human heuristic employed.

The effectiveness of an active learning (AL) algorithm is typically measured as the difference between the performance of the AL algorithm and that of random sampling. Following this approach, the increase in effectiveness can be computed using the formula below:


$$\begin{array}{l} \text{Increase in effectiveness}\\ =\frac{Avg.AUC_{withdropout}-Avg.AUC_{withoutdropout}}{Avg.AUC_{withoutdropout}-Avg.AUC_{Random}} \end{array}$$

The effectiveness of the information density algorithm increased by 113%, while for entropy sampling, this improvement was more than three times greater. Similarly, the proposed mechanism resulted in a 78% improvement when the TTB heuristic was used to provide labels, and this increase was sixfold when the Fast and Frugal Tree heuristic was applied.

Although the frequency of cases where the proposed mechanism showed an improvement was relatively consistent across active learning algorithms and labeling strategies, the magnitude of the improvement was notably higher under entropy sampling and the Fast and Frugal Tree conditions. This further emphasizes the potential benefits of using these algorithms in such scenarios.

## 5 Limitations and scope of future work

This study is conducted under specific conditions and assumptions, such as linear separability and uniform distribution of decision boundaries. While these constraints limit the theoretical generalizability of the proposed mathematical formulation, they serve to motivate future work aimed at relaxing these assumptions. Importantly, despite these theoretical limitations, the proposed mechanisms have been evaluated on well-established real-world datasets and have demonstrated significant improvements in performance across a majority of scenarios. This empirical success suggests the potential applicability of our methods in broader contexts.

However, the current study focuses on modeling the fast and frugal heuristics that annotators may use while labeling, and designs dropout-based mechanisms to address their effects. We deliberately avoid human-in-the-loop experiments in this work to maintain scope and control, as introducing human participants would add considerable stochasticity due to the unpredictability of heuristic usage. In practice, it is often impossible to know which specific heuristic an annotator uses for each query, given the wide range of possible strategies.

Future work can explore incorporating human-in-the-loop experiments to capture this uncertainty in heuristic behavior and to design robust mechanisms that generalize across diverse human annotation strategies. Moreover, the applicability of the proposed dropout mechanisms in more complex scenarios, such as multi-class classification or cases where annotators employ highly efficient heuristics, remains an open question. In such settings, the mechanisms may not yield similar gains and could potentially lead to a decline in active learning performance, thereby providing another avenue for further investigation.

## 6 Conclusion

Building on the intuitive understanding that human-provided labels for active learning can sometimes be influenced by biases, we model the oracle's behavior using fast-and-frugal heuristics. To address the challenges posed by such heuristics, this study introduces a novel drop-out mechanism that shifts the focus of active learning from querying strategies to directly influencing the labeling process. By selectively presenting attributes to the oracle, this mechanism effectively reduces the likelihood of incorrect labels, thereby improving overall label quality.

The mathematical framework derived for the proposed mechanism enables the computation of the probability of incorrect labeling in a two-dimensional space when one attribute is used. This formulation not only validates the design of the mechanism but also demonstrates its potential to enhance active learning performance significantly, as evidenced by experimental results. The integration of this approach leads to a notable improvement in the quality of labeled data and the efficiency of the learning process.

Looking ahead, this study opens new directions for advancing active learning by exploring extensions of the derived framework to higher-dimensional spaces. It also highlights the potential for deeper integration of human and algorithmic decision-making, paving the way for innovative strategies in labeling tasks and adaptive learning systems.

## Data Availability

The original contributions presented in the study are included in the article and further inquiries can be directed to the corresponding author. The codeset required to replicate this study is available at https://github.com/SriramML/A-Drop-out-mechanism-for-AL.git.
